# Advancing Personalized Medicine in Psychiatry: A Descriptive Pilot Study Integrating Pharmacogenetics and Pharmacokinetics in Long-Acting Antipsychotic Treatment

**DOI:** 10.3390/pharmaceutics18080958

**Published:** 2026-08-04

**Authors:** Almudena Gil-Rodriguez, Sheila Recarey-Rama, María Vidal-Millares, Francisco José Toja-Camba, María Tajes, Verónica Prado-Robles, María José Durán-Maseda, Manuela Pérez García, Ana Rodríguez-Viyuela, Patricia Sánchez-Fariña, María Jesús Abeledo-Lameiro, Mario Páramo, Fernando Facal, Manuel Arrojo Romero, Almudena Diaz Pereira, Cristina Mondelo-García, Anxo Fernández-Ferreiro, Angel Carracedo, Olalla Maroñas

**Affiliations:** 1 Grupo de Farmacogenómica y Descubrimiento de Medicamentos (GenDeM), Instituto de Investigación de Santiago de Compostela (IDIS), 15706 Santiago de Compostela, Spain; almudena.gil@usc.es (A.G.-R.); sheila.recarey.rama@usc.es (S.R.-R.); patricia.sanchez@usc.es (P.S.-F.); 2 Grupo de Medicina Xenómica, Centro Singular de Investigación en Medicina Molecular y Enfermedades Crónicas (CiMUS), Universidade de Santiago de Compostela, 15782 Santiago de Compostela, Spain; anarovi15@gmail.com (A.R.-V.); angel.carracedo@usc.es (A.C.); 3 Servicio de Psiquiatría, Complejo Hospitalario Universitario de Santiago de Compostela, Servizo Galego de Saúde (SERGAS), 15706 Santiago de Compostela, Spain; maria.vidal.millares@sergas.es (M.V.-M.); veronica.prado.robles@sergas.es (V.P.-R.); maria.jose.duran.maseda@sergas.es (M.J.D.-M.); manuela.perez.garcia@sergas.es (M.P.G.); mario.paramo.fernandez@sergas.es (M.P.); fernando.facal.molina@sergas.es (F.F.); manuel.arrojo.romero@sergas.es (M.A.R.); 4 Departamento de Psiquiatría, Radiología, Salud Pública, Enfermería y Medicina, Universidade de Santiago de Compostela, 15782 Santiago de Compostela, Spain; 5 Servicio de Farmacia, Complejo Hospitalario Universitario de Santiago de Compostela, Servizo Galego de Saúde (SERGAS), 15706 Santiago de Compostela, Spain; kikotoja@gmail.com (F.J.T.-C.); crismondelo1@gmail.com (C.M.-G.); anxordes@gmail.com (A.F.-F.); 6 Grupo FarmaCHUSLab, Instituto de Investigación de Santiago de Compostela (IDIS), 15706 Santiago de Compostela, Spain; 7 Servicio de Psiquiatría, Complejo Hospitalario Universitario de Ourense, Servizo Galego de Saúde (SERGAS), 32005 Ourense, Spain; maria.tajes.alonso@sergas.es; 8 Grupo de Genética, Instituto de Investigación de Santiago de Compostela (IDIS), 15706 Santiago de Compostela, Spain; 9 Fundación Pública Galega de Medicina Xenómica (FPGMX), Servizo Galego de Saúde (SERGAS), 15706 Santiago de Compostela, Spain; maria.jesus.abeledo.lameiro@sergas.es; 10 Grupo de Genética Psiquiátrica, Instituto de Investigación de Santiago de Compostela (IDIS), 15706 Santiago de Compostela, Spain; 11 Departamento de Salud Mental, Autoridad Sanitaria Regional, Xunta de Galicia, 15003 A Coruña, Spain; almudena.diaz.pereira@sergas.es; 12 Centro de Investigación Biomédica en Red en Enfermedades Raras (CIBERER), Instituto de Salud Carlos III, 28029 Madrid, Spain

**Keywords:** biomarkers, program evaluation, genetic variation, drug monitoring, clinical decision support systems, genotype, psychopharmacology, drug interactions

## Abstract

**Background:** Personalized precision medicine adapts therapeutic strategies to individual characteristics. In psychiatry, suboptimal outcomes with antipsychotics are often related to adverse drug reactions, poor adherence and therapeutic failure. Pharmacogenetics, particularly CYP2D6 genotyping, can improve clinical outcomes through genotype-guided therapy. This study aimed to design and implement a pharmacogenomic and pharmacokinetic testing program for long-acting injectable (LAI) antipsychotics within the Galician Health Service to enhance personalized psychiatric care. **Methods:** The pilot program encompasses pharmacogenetic and pharmacokinetic testing. Inclusion criteria were broad, covering patients initiating or receiving LAI antipsychotic therapy, as well as those with prior adverse reactions in order to explore scenarios where pharmacogenetic and/or pharmacokinetic data could help with clinical decisions. Structured workflows, interdisciplinary training and integration of results into the electronic health record supported implementation. A pharmacogenetic panel was specifically designed for psychiatric care, targeting clinically relevant variants in CYP2D6, CYP3A4 and ABCB1. **Results:** A total of 540 patients were included, with primary testing reasons being clinical follow-up (54.6%) and oral-to-LAI transition (38.5%). The CYP2D6 phenotypes were 55% normal, 34% intermediate, 6.3% poor and 4.3% ultrarapid metabolizers. Atypical metabolism was observed in 6.7% of patients for CYP3A4 and in over half for ABCB1. Plasma drug levels were within the therapeutic range for most patients, though some measurements were above or below expected values. **Conclusions:** This pilot demonstrates a scalable, evidence-based approach to precision psychiatry for LAI antipsychotics, integrating pharmacogenetic and pharmacokinetic testing into routine care. The framework facilitates genotype-guided decision-making and supports broader adoption of pharmacogenomics in psychiatric practice.

## 1. Introduction

Personalized Precision Medicine (PPM) represents a major transformation of healthcare by personalizing preventive, diagnostic and therapeutic strategies according to the individual characteristics of each patient [1,2]. The response to a drug is a multifactorial process that requires consideration of factors associated with the disease, the drug itself and the individual. Physiological, environmental and clinical factors influence drug response, together with pharmacogenetics playing a key role in terms of efficacy or adverse reactions [3,4,5,6,7,8,9,10,11,12]. Antipsychotics are the primary approach for managing schizophrenia, a severe and highly heterogeneous mental disorder affecting about 0,32% of the global population [13]. Approximately 20–50% of patients develop treatment-resistant schizophrenia despite receiving adequate antipsychotic therapy, highlighting the considerable interindividual variability in treatment response [14]. Furthermore, medication non-adherence affects nearly 50% of patients with schizophrenia and represents one of the leading causes of relapse, rehospitalization and poor long-term outcomes [15,16]. In addition, adverse drug reactions—including extrapyramidal symptoms, metabolic disturbances, sedation and hyperprolactinemia—together with psychosocial factors such as poor insight, negative attitudes toward medication and substance abuse, are major contributors to treatment discontinuation and reduced therapeutic effectiveness [15,17]. However, these issues have been partially addressed by long-acting injectable (LAI) antipsychotics, which improve adherence and potentially lead to better outcomes [18]. In addition to adherence-related challenges, optimizing the dose of antipsychotic treatment is a critical determinant of therapeutic response and long-term outcomes. In current clinical practice, dose adjustments are frequently based on trial-and-error approaches and clinical experience, which may expose patients to adverse reactions, prolong recovery time, and compromise treatment effectiveness [19,20,21]. Interindividual variability in pharmacokinetics—including differences in drug absorption, distribution, metabolism and excretion—can result in markedly different plasma exposures among patients receiving the same dose, directly influencing both efficacy and safety.

Antipsychotics are mainly metabolized by cytochrome P450 enzymes, particularly CYP2D6. This enzyme plays a crucial role in the biotransformation and clinical response of about 20% of commonly used drugs, including antidepressants, antihypertensives and anticancer agents [22]. Therapeutic drug monitoring (TDM) has emerged as a valuable strategy to support dose individualization. Maintaining antipsychotic plasma concentrations within established therapeutic ranges improves clinical outcomes, as shown in patients treated with clozapine, who achieved an 84% improvement when plasma levels remained within the therapeutic range [23], while supratherapeutic concentrations of risperidone increased the incidence of extrapyramidal adverse effects by 35% [24]. In addition, TDM allows the detection of partial non-adherence—affecting up to 30% of patients—and pharmacological interactions due to polypharmacy, which is common in psychiatric populations [25]. Reference therapeutic ranges for frequently prescribed antipsychotics are defined in the AGNP Consensus Guidelines for Therapeutic Drug Monitoring in Neuropsychopharmacology [26].

Drug regulatory agencies and pharmacogenetic consortia have issued clinical guidelines that promote treatment personalization based on genetic profiles [27,28,29,30]. However, the successful adoption of pharmacogenetics depends on national strategies ensuring quality, equitable access and sustainability. Preventive genetic information obtained prior to the initiation of treatment enables safer and more personalized clinical decision-making, as demonstrated by DPYD genotyping in patients scheduled to receive fluoropyrimidines, thereby reducing the risk of severe toxicities [6]. In contrast, in the field of psychopharmacology, pharmacogenetic testing is typically applied reactively—i.e., after the patient has experienced an adverse reaction or therapeutic failure—with the goal of retrospectively identifying and addressing the issue. Although personalized medicine initiatives are growing worldwide, clinical implementation varies according to local infrastructure and policies and the reactive approach remains the most commonly used in routine clinical practice [31,32,33].

In the United States, the Food and Drug Administration (FDA) and organisms such as the Clinical Pharmacogenetics Implementation Consortium (CPIC) and the Pharmacogenomics Research Network (PGRN) create freely available, evidence-based gene–drug clinical guidelines [34]. Additionally, the PGRN promotes the standardization and sharing of real-world implementation strategies, respectively [35]. Various initiatives focused on the translation of pharmacogenetics into routine clinical practice have been developed. Projects such as eMERGE-PGx or IGNITE have focused on preemptive pharmacogenetic (PGx) testing and data integration [36,37,38,39]. Other programs such as PG4KDS at St. Jude Children’s Research Hospital or the RIGHT project at the Mayo Clinic, among others, have focused on clinical workflows with electronic health record support [40,41,42,43,44]. Finally, initiatives such as INGENIOUS or the 1200 Patients Project and the All of Us Research Program, aim to evaluate the real-world impact of pharmacogenomics on outcomes and costs [45,46,47].

In Asia and the Middle East, various countries like South Korea (1K Korean Project), Singapore (Singapore 10K Genome Project), Qatar (QPGx-CARE initiative) and Lebanon are either leading pilot studies or developing guidelines with the aim of advancing pharmacogenomics research and clinical use through regional and national initiatives [48,49,50,51,52,53].

In Europe, the European Medicines Agency (EMA) and the Dutch Pharmacogenetics Working Group (DPWG) develop pharmacogenetic recommendations. Additionally, the DPWG publishes therapeutic guidelines that serve as a reference for the implementation of pharmacogenetics and support clinical practice by integrating these recommendations into electronic prescribing and monitoring systems [54,55]. Complementing these efforts, the European Society of Pharmacogenomics and Personalized Therapy (ESPT) promotes research and education toward the adoption of pharmacogenetics across Europe [56]. The PREPARE study, led by the Ubiquitous Pharmacogenomics Consortium (U-PGx), performs genotype-guided therapy, reducing adverse drug reactions, and analyzes the feasibility of implementing these analyses across different healthcare systems [57,58,59,60]. Another initiative, the PSY-PGx project, has focused on the development of a genotyping approach for individualized pharmacotherapy in order to improve treatment outcomes in patients with anxiety, depression and psychotic disorders [61,62]. With the aim of facilitating access to and reuse of health data for research purposes while ensuring interoperability among entities, significant efforts have been invested in developing the European Health Data Space Regulation [63]. In line, the 1+ Million Genomes Initiative (1+ MG) supported by national counterparts known as “National Mirror Groups”—with a group devoted to pharmacogenomics—aims to achieve the sequence of one million genomes [64].

In Spain, different strategies have been developed through European funds, the 5P Plan and the Vanguard Health PERTE to promote coordination, collaboration and innovation in PPM including collaborative research projects through the Infrastructure for Precision Medicine associated with Science and Technology (IMPaCT), led by the Carlos III Health Institute [65]. In 2023, the approval of a Common Genetics Portfolio of Services of the National Health System promoted the homogeneous implementation of genetic tests and ensured equity across the country [66,67]. This portfolio encompasses a specific catalog for pharmacogenetics covering 22 drugs and 12 genes. In line with these efforts, the Spanish Agency for Medicines and Medical Devices (AEMPS) launched a database of pharmacogenomic biomarkers that compiles clinically relevant associations between drugs and genetic biomarkers [68]. Finally, the Spanish Society of Pharmacogenetics and Pharmacogenomics (SEFF) is actively involved in developing recommendation documents in Spanish and disseminating scientific knowledge [69].

In recent years, an increasing number of initiatives focused on the implementation of PPM into clinical care have been launched [70,71]. Projects such as MedeA in Extremadura [72], PriME-PGx in Madrid [73] or NAGEN and PharmaNAGEN in Navarra [74], incorporate pharmacogenetics into routine practice, aiming to improve patient outcomes and optimize therapeutics strategies. In parallel health plans have been developed, with Catalonia and the Basque Country being the clearest and most well-defined related to PPM [75,76]. According to the Roche Institute report 2018 [77], Galicia and Castilla y León were the first regions in Spain to develop formal PPM implementation plans. In Castilla y León, as part of the Strategic Plan for Personalized Medicine, the five-step precision medicine model (5SPM), developed by the Reference Unit in Pharmacogenetics and Precision Medicine in the Salamanca Hospital Complex, serves to guide the application of pharmacogenomics in cases of therapeutic failure or serious adverse effects in routine clinical care [78,79,80]. In Galicia, for the last 22 years, the Public Galician Foundation of Genomic Medicine (FPGMX) has been the reference center for genomic medicine for all Galician hospitals [9,81]. The FPGMX provides a comprehensive range of clinical genetic services, including genetic counseling, prenatal diagnosis, oncohematology and solid tumor oncology, hereditary diseases and pharmacogenetics as well as research activities. In 2020, the Galician healthcare system and the FPGMX launched the Implementation Plan for Pharmacogenomics in Psychiatry, an initiative focused on the clinical implementation of pharmacogenetic and pharmacokinetic testing for LAI antipsychotics, as part of the Galician Post–COVID-19 Mental Health Plan [82]. This pioneering initiative addresses the need to integrate PPM into clinical practice for psychiatric patients as a complementary tool in therapeutic decision-making, with the ultimate goal of incorporating it into the Galician Health Service portfolio. This article focuses on the development of the Pharmacogenetics Implementation Plan, covering the essential aspects for implementation (including participant training, workflows, analyses and the integration of results) and providing a comprehensive approach to incorporate pharmacogenetics and pharmacokinetics into clinical care.

## 2. Materials and Methods

### 2.1. Framework for the Pilot Program

This study is a descriptive implementation study reporting the design, implementation and preliminary results of the Pharmacogenetics Implementation Pilot Program in Psychiatry, developed within SERGAS.

The Implementation Plan for Pharmacogenetics in Psychiatry is a pilot program included in the post-COVID-19 Mental Health Plan of Galicia developed by SERGAS, the FPGMX and the Hospital Pharmacy Service [82]. One of the initiatives of this plan is focused on the clinical implementation of pharmacogenetic and pharmacokinetic testing for LAI antipsychotics. The main goal is to integrate these personalized medicine tools into routine psychiatric care to reduce hospitalizations, minimize treatment-related adverse effects, and enhance patient well-being. Inclusion criteria encompassed patients initiating treatment with aripiprazole, risperidone or paliperidone depot formulations, patients undergoing oral-to-LAI transition, those requiring therapeutic drug monitoring as part of routine clinical practice—including suspected adverse drug reactions, inadequate clinical response, suspected non-adherence, dosage adjustments, potential drug–drug interactions or other situations in which plasma drug concentrations could support clinical decision-making—and patients receiving concomitant medications with potential interactions with the antipsychotic [82]. In accordance with consensus guidelines for therapeutic drug monitoring, the therapeutic range for risperidone and 9-OH-risperidone (paliperidone), as well as for their combined concentration, is 20–60 ng/mL. In the case of aripiprazole, the recommended therapeutic range is 100–350 ng/mL, whereas the combined concentration of aripiprazole plus dehydroaripiprazole ranges from 150 to 500 ng/mL [26].

As previously stated, antipsychotics are mainly metabolized by CYP2D6; therefore, variants in this gene may result in altered enzymatic activity and potentially lead to adverse effects [83]. Although to a lesser extent, CYP3A4 is also involved in the metabolism of antipsychotics [84,85]. Finally, it is worth mentioning that the efflux transporter P-glycoprotein (P-gp) plays a role in regulating xenobiotic transportation across the blood–brain barrier. Variants altering the functionality of this transporter may limit xenobiotic penetration and consequently reduce its therapeutic effects [86].

In light of these genetic influences, regulatory agencies have issued specific dosage recommendations based on the metabolic status of the patient. In the case of aripiprazole, AEMPS, the EMA and the FDA have issued a reduction in the initial dose for patients with a reduced enzymatic activity of CYP2D6 (poor metabolizers). Furthermore, additional reductions have been recommended for patients concomitantly treated with CYP3A4 inhibitors. For risperidone, the AEMPS pharmacogenetic biomarker database reports that, regarding risperidone and CYP2D6 metabolizers, ultrarapid metabolizers exhibit lower risperidone concentrations and higher 9-hydroxy-risperidone (paliperidone) levels than slow metabolizers; however, the active antipsychotic fraction (risperidone + 9-hydroxy-risperidone) displays a similar pharmacokinetic profile in both groups after single or multiple doses. The EMA and FDA also mention the role of CYP2D6 in the metabolism of risperidone; however, no differences between poor and extensive metabolizers have been stated. According to the AEMPS, in vitro studies indicate that paliperidone metabolism involves the CYP2D6 and CYP3A4 enzymes and that the compound also serves as a substrate for the P-gp protein. In contrast, both the EMA and FDA state that paliperidone is not significantly affected by these enzymes or by medications that inhibit them.

### 2.2. Involved Translational Units

For more than 25 years, the FPGMX has centralized 80% of the genetic analyses of SERGAS which integrates, coordinates, and organizes all regional public hospitals, services and health facilities in the region. The SERGAS covers approximately 2.7 million inhabitants organized in seven Integrated Management Structures or Health Areas which share a unique electronic medical record—called IANUS—that compiles all clinical information about patients [87]. The Pharmacogenetics Unit of the FPGMX is devoted to the development of healthcare pharmacogenetic analyses to be integrated into clinical practice, as well as to the development of research projects in the area [88]. Every year, more than 1500 tests are received from hospitals throughout Galicia, especially from areas such as oncology, psychiatry and cardiology [3,7,9,10,12,54,89,90]. In terms of quality assurance, it is worth highlighting that the FPGMX participates annually in European external quality assessments and also leads the development of Pharmacogenetic Proficiency Testing in Spain [91,92].

The Hospital Pharmacy Service of the Clinical University Hospital of Santiago, part of the Integrated Management Division of Santiago de Compostela y Barbanza, provides pharmaceutical care to both inpatients and outpatients and covers a population of approximately 450,000 people. In addition, this service is responsible for the pharmacokinetic analyses of the Santiago Health Area [93].

The Plan encompasses all healthcare resources of the Santiago Health Area Psychiatric Service, including acute and long-term psychiatric hospitalization units, outpatient Mental Health Units and intermediate care services such as Home Hospitalization, the First Psychotic Episodes Program, the Care Continuity Team and the Day Hospital.

This diversity reflects the broad scope of the pharmacogenetics service within mental health, covering both patients experiencing severe psychiatric episodes requiring immediate intervention and patients needing extended care and ongoing management. It is worth mentioning that these pharmacogenetic analyses are fully covered by the Galician public health system (SERGAS) at no cost to the patient, ensuring equitable access to personalized medicine.

### 2.3. Early Steps and Dissemination

**Figure 1:**
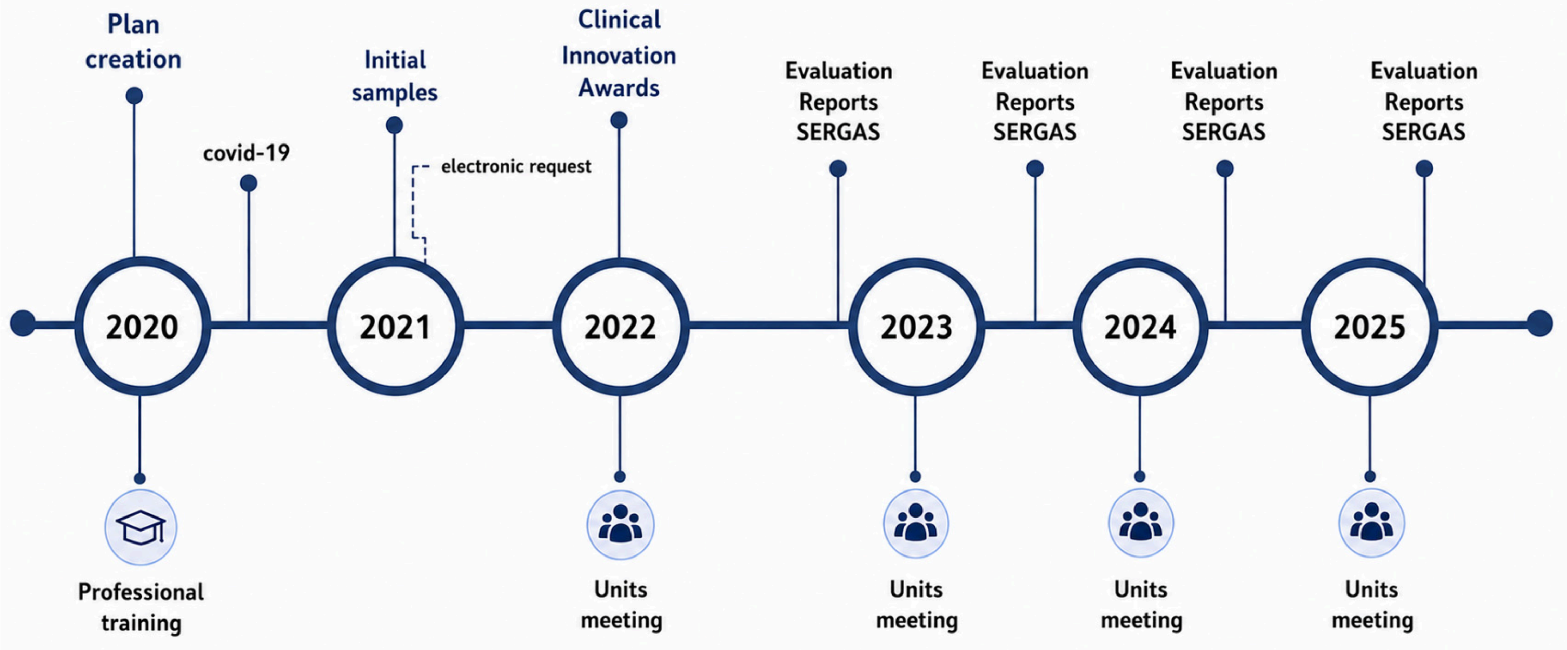
Timeline of the pilot project. Main stages and key points from the planning phase in 2020 to the end of 2025.

The plan was developed in 2020, but its launch was postponed until 2021 due to the global COVID-19 pandemic. Early steps involved coordination meetings between the translational units, alongside the Mental Health Units. Training sessions in pharmacogenetics and pharmacokinetics, in collaboration with the Galician Agency for Health Knowledge (ACIS) were designed for all prescribing professionals participating in the Plan. Additionally, regular meetings have been maintained with the psychiatrists in order to solve doubts and monitor progress. It is worth highlighting that in 2022, the Implementation Plan for Pharmacogenetics in Psychiatry for LAI antipsychotics was awarded for Clinical Innovation in the Clinical Program category at the XXV National Congress of Psychiatry and Mental Health in Spain, through the work presented entitled *Clinical Implementation of Pharmacokinetics and Pharmacogenetics of Depot Antipsychotics*. Different evaluation reports have been submitted to SERGAS, with the purpose of reviewing the plan, assessing its implementation and outcomes, as well as identifying emerging needs for improvement (Figure 1).

### 2.4. Established Workflows

**Figure 2:**
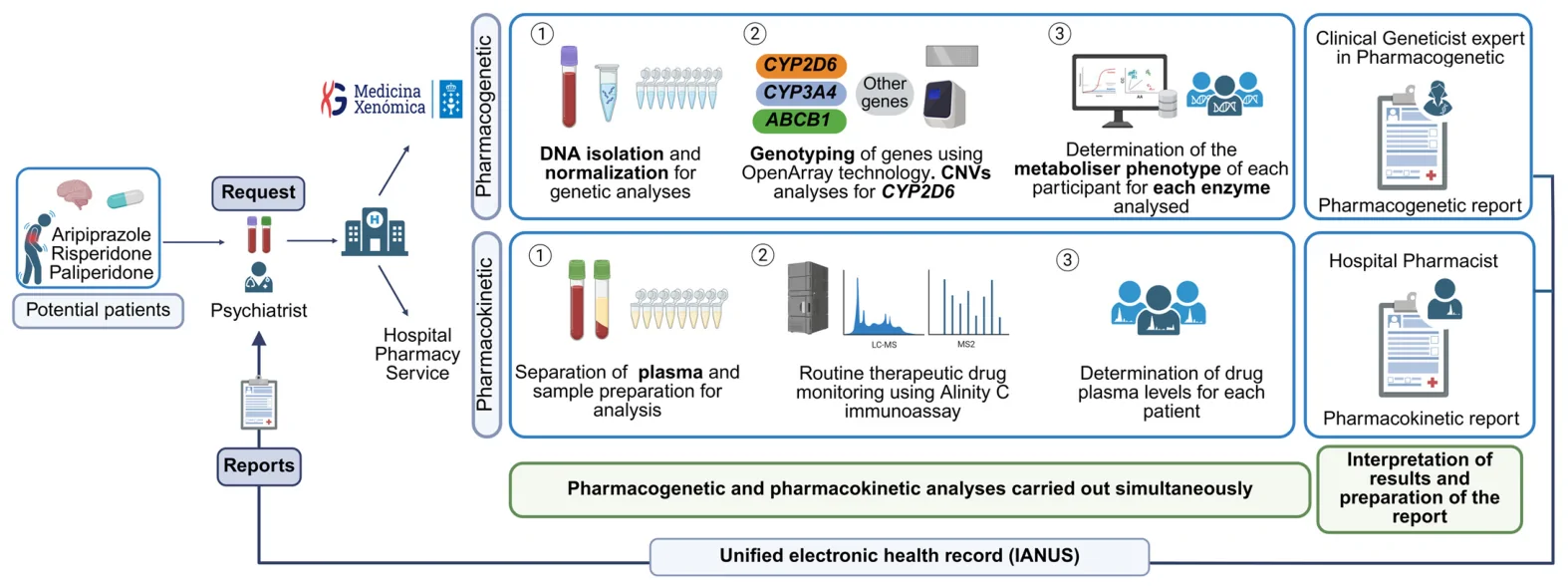
Sample request, analysis workflow and electronic reports. Created in BioRender. Carracedo, Á. (2026) https://BioRender.com/m6vsct3.

The previously established care workflows of both units were used for the development of the Plan. Two tubes of blood (EDTA) were collected from each patient, one for pharmacogenetics and the other for pharmacokinetics. Initially, test requests were performed on paper. However, within the framework of the Plan, a specific electronic request has been developed, allowing psychiatrists to conveniently order pharmacogenetic and pharmacokinetic tests. These electronic requests are performed directly through the electronic healthcare record, improving both accessibility and efficiency of the process. Once the analyses have been completed, two separate reports with the results are uploaded into the electronic healthcare record (IANUS) of the patient (Figure 2). Estimated turnaround times for the analyses were established with the Units. Regular meetings have been held with psychiatrists, enabling continuous communication and strengthening interdisciplinary collaboration among psychiatrists, geneticists and pharmacists throughout the entire implementation process.

### 2.5. Pharmacogenetic Panel Development

Following a thorough review of publications and pharmacogenetic databases in the field of psychiatry, a pharmacogenetic panel was developed including *CYP2D6*, *CYP3A4* and *ABCB1* (Table 1), together with other relevant genes. For *CYP2D6*, a list of reduced and non-functional alleles, together with regions to interrogate copy number variation, was selected. For the *CYP3A4* gene, alleles *20 and *22, both encoding for reduced enzyme function, were included. For the *ABCB1* gene, despite the limited strength of evidence, variants reported to be associated with reduced P-glycoprotein function at positions c.3435T>C, c.2677T>G/A and c.1236T>C were included [86].

It is worth highlighting that our panel, which was designed in 2020, already incorporates all the alleles in the genes included in the Official Service Portfolio for Pharmacogenetics, which was published by the Ministry of Health in 2023 [66], as well as additional alleles of interest.

### 2.6. Pharmacogenetic Analysis

Pharmacogenetic analyses at the FPGMX are performed by real-time PCR in the QuantStudio 12K Flex System (Applied Biosystems by Life Technologies, Waltham, MA, USA) and QuantStudio 5 System (Applied Biosystems by Life Technologies, Waltham, MA, USA) platforms following the manufacturer’s specifications for TaqMan technology. Both single-nucleotide polymorphisms (SNPs) and insertions/deletions (indels), as well as copy number variation for *CYP2D6*—essential for assigning the patient’s metabolizer status—have been analyzed. Two software, QuantStudio™ 12K Flex Real-Time PCR Software and Taqman^®^ Genotyper Software, were used to analyze results. Positive and negative controls were used in the analyses.

*CYP2D6* genotype-to-phenotype translation was performed following CPIC recommendations. Diplotypes were assigned an activity score and classified into the corresponding metabolizer phenotype (poor, intermediate, normal, or ultrarapid metabolizer) [94].

### 2.7. Pharmacokinetic Monitoring

Pharmacokinetic (PK) determinations of risperidone, paliperidone and aripiprazole have been carried out by the Hospital Pharmacy Service. For these analyses, blood samples must be collected immediately prior to the administration of the monitored drug, in accordance with the AGNP Consensus Guidelines for Therapeutic Drug Monitoring in Neuropsychopharmacology [26]. Depending on the formulation and half-life of the antipsychotic, the minimum number of doses required before sampling varied to ensure steady-state conditions (Table 2).

The metabolism of risperidone involves the CYP2D6 enzyme, which is primarily responsible for the formation of 9-OH-risperidone (9-OH-R, paliperidone), the main active metabolite [86]. In accordance with consensus guidelines on monitoring, the therapeutic range for the combined levels of risperidone plus 9-OH-risperidone (paliperidone) is between 20 and 60 ng/mL. The therapeutic range for paliperidone palmitate is also between 20 and 60 ng/mL [26]. In the case of aripiprazole, its metabolism into the active metabolite dehydroaripiprazole (DHA) is primarily mediated by the CYP2D6 and CYP3A4 enzymes [95]. In accordance with consensus guidelines on monitoring, the therapeutic range for aripiprazole is 100–350 ng/mL and for aripiprazole plus DHA is 150–500 ng/mL [26].

### 2.8. Pharmacokinetic Analysis

The quantification of the concentration of antipsychotics was carried out with the analyzer Alinity C (Abbott Laboratories). This equipment implements photometric and potentiometric detection technologies for the quantitative measurement of analytes in human serum, plasma, urine, cerebrospinal fluid, hemolysate, and whole blood. The photometric assays used for the determination of the active moieties of aripiprazole and risperidone on the Alinity C analyzer employ a tungsten lamp, a diffraction grating, and a silicon photodiode detector, with measurements performed at a wavelength of 604 nm.

For aripiprazole determination, the MyCare Psychiatry Kit for total aripiprazole (Saladax Biomedical, Bethlehem, PA, USA) was used to measure the concentration of the active moiety, defined as the sum of the parent drug and its pharmacologically active metabolite (aripiprazole plus dehydroaripiprazole). Similarly, for risperidone, the MyCare Psychiatry Kit for total risperidone (Saladax Biomedical, Bethlehem, PA, USA) was used for the determination of the active moiety, corresponding to the combined concentration of risperidone and its active metabolite paliperidone in whole blood. Both immunoassays are based on a competitive binding principle between the drug present in the patient’s sample and drug conjugates (or calibrators and controls provided in the kit) for binding to drug-specific antibodies covalently bound to nanoparticles. The resulting nanoparticle aggregation is monitored spectrophotometrically using clinical chemistry analyzers such as Alinity C.

Cross-reactivity for both assays was evaluated by the manufacturer using more than 150 drugs, as well as in the presence of potential interfering substances including rheumatoid factor, lipids, and hemolyzed samples. The lower and upper limits of quantification for the aripiprazole kit are 45 ng/mL and 1000 ng/mL, respectively, whereas for the risperidone kit they are 16 ng/mL and 120 ng/mL. Because the Alinity C analyzer provides values only for the active moiety—aripiprazole plus dehydroaripiprazole in the case of aripiprazole, and risperidone plus paliperidone in the case of risperidone—ultra-high-performance liquid chromatography–tandem mass spectrometry (UHPLC-MS/MS) on a Xevo TQD^®^ triple quadrupole mass spectrometer (Waters, Milford, MA, USA) was used when quantification of both components separately was required [96,97].

### 2.9. Statistical Analysis

Given the descriptive nature of this implementation study, data were summarized using descriptive statistics. Continuous variables are presented as mean ± standard deviation (SD), whereas categorical variables are expressed as frequencies and percentages.

## 3. Results

### 3.1. Demographics

A total of 540 requests have been received through the Pilot Program from 2021 to October 2025. The mean age of the patients was 47.18 ± 14.4 years. The study population included 57.4% males (*n* = 310) and 42.6% females (*n* = 230). Regarding treatment with aripiprazole, risperidone, and paliperidone, most patients received only one of these agents: paliperidone 40.74% (*n* = 220), aripiprazole 35.93% (*n* = 194) and risperidone 17.96% (*n* = 97), regardless of any other concomitant medications. A total of 5.4% of patients (*n* = 29) received a combination of two of these antipsychotics, independent of other treatments. Considering each drug’s involvement in polytherapy, aripiprazole was present in 65.5% (*n* = 19) of patients receiving polytherapy in combination with risperidone in a 41.4% (*n* = 12) and paliperidone in a 24.1% (*n* = 7). Similarly, risperidone was present in 75.9% (*n* = 22) of patients in combination with aripiprazole (41.4%, *n* = 12) and paliperidone (34.5%, *n* = 10). Finally, paliperidone was present in 58.6% (*n* = 17) of patients, in 24.1% (*n* = 7) in combination with aripiprazole and in 34.5% (*n* = 10) with risperidone. The most common indication was clinical follow-up of patients receiving LAI treatment, accounting for 54.63% (*n* = 295) of patients, followed by transition from oral to LAI therapy in 38.52% (*n* = 208) and finally suspected adverse drug reactions in 6.85% (*n* = 37).

### 3.2. Drug Prescriptions

**Figure 3:**
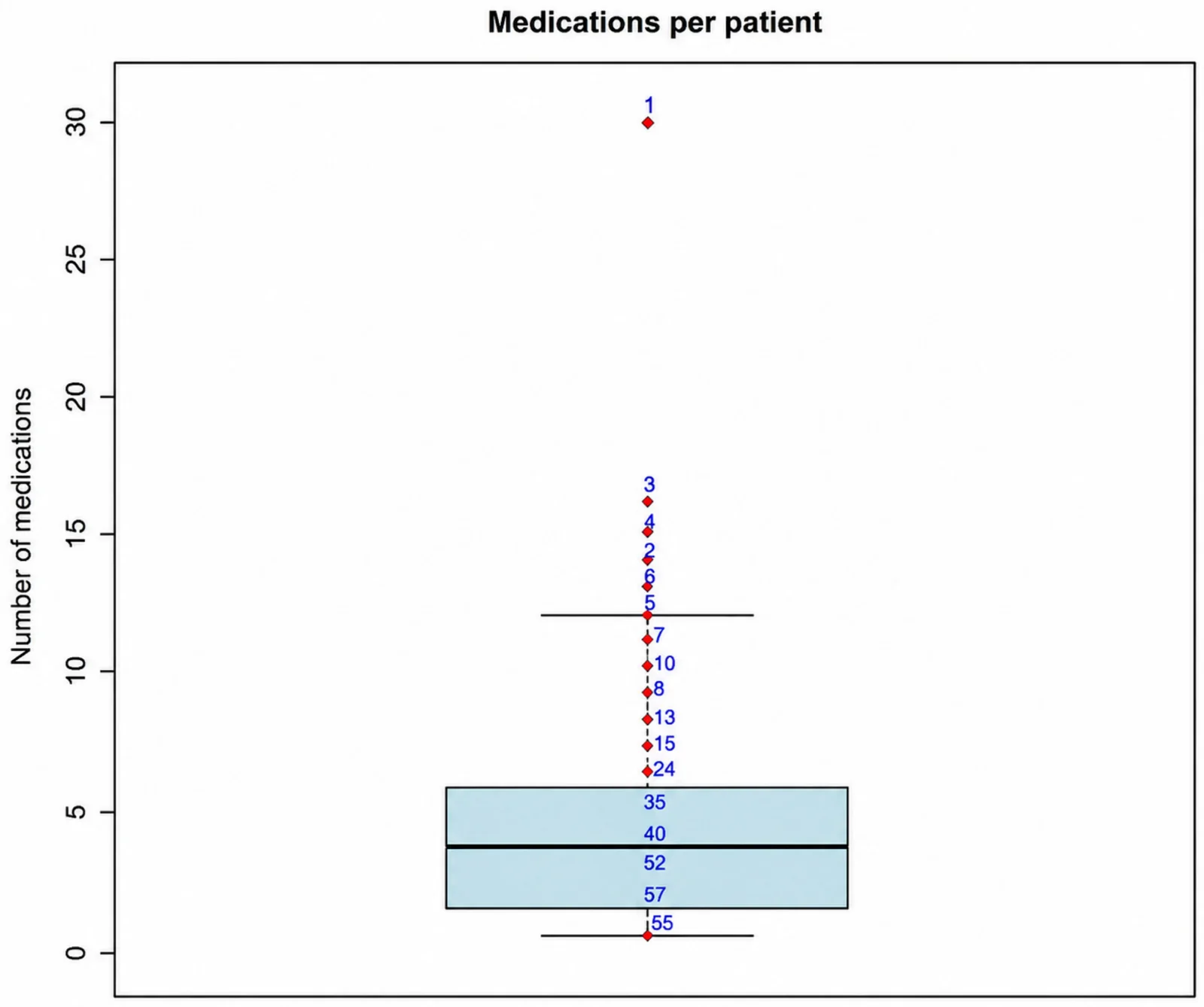
Distribution of the Number of Medications per Patient. Boxplot showing the distribution of the number of medications prescribed per patient.

Among the 540 patients analyzed, data on concomitant medication were successfully obtained for 333 individuals. Figure 3 illustrates the distribution of the number of concomitant drugs prescribed per patient. Based on the distribution data, the number of concomitant drugs prescribed per patient ranged from 1 to 30, with 80% of the patients receiving between one and six medications. The median number of concomitant medications was 4. A 3.6% of patients exhibited high levels of polypharmacy, with more than twelve concomitant drugs, including one extreme case with thirty. This distribution indicates that while the majority of patients are managed with a moderate number of concomitant therapies, a subset experiences a substantial pharmacological burden likely associated with greater clinical complexity and comorbidity (Figure 3).

**Figure 4:**
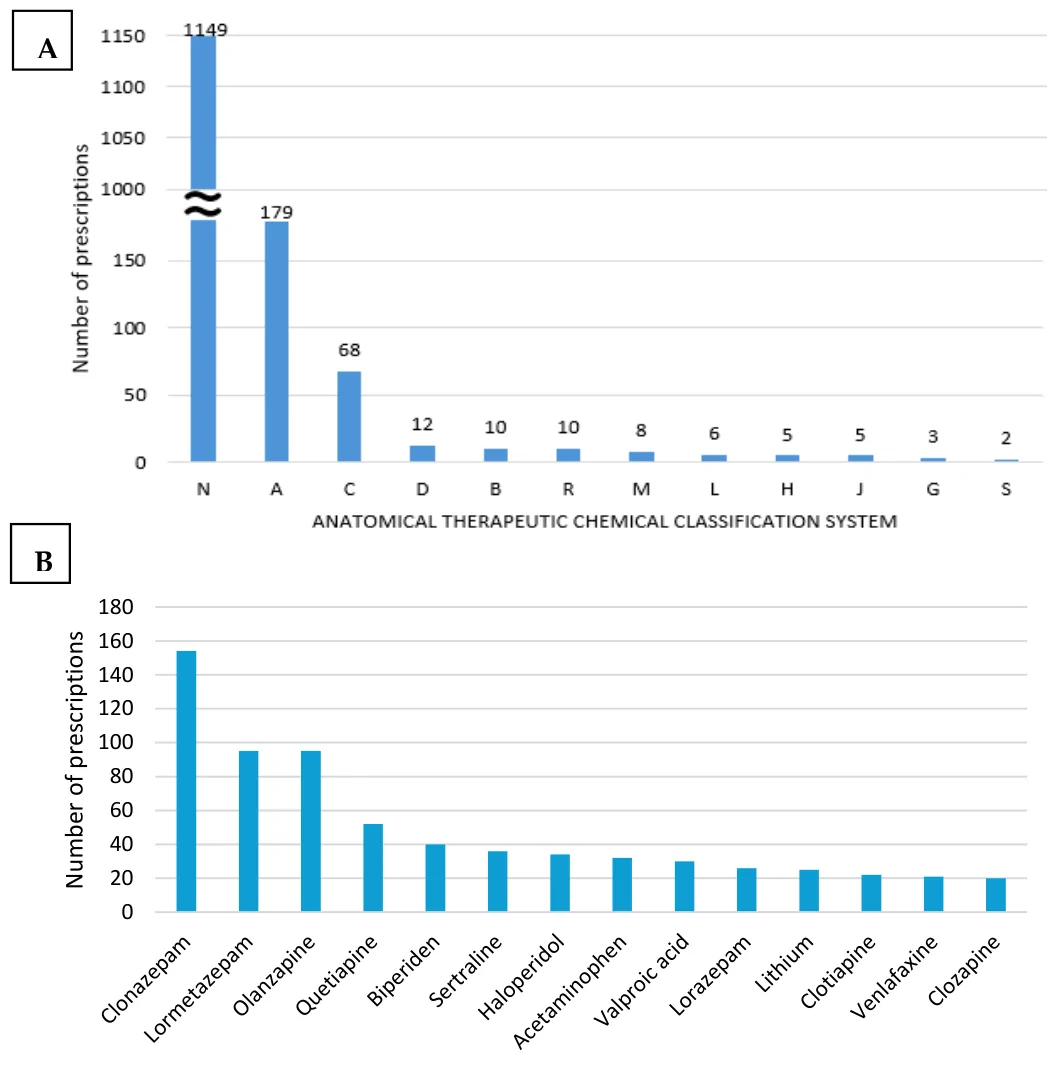
(**A**) Distribution of Prescribed Drugs by Anatomical Therapeutic Chemical (ATC) Classification. A: Digestive Tract and Metabolism; B: Blood and Blood-Forming Organs; C: Cardiovascular System; D: Dermatological; G: Genitourinary System and Sex Hormones; H: Systemic Hormonal Preparations (excluding sex hormones); J: Anti-infectives for Systemic Use; L: Antineoplastic and Immunomodulating Agents; M: Musculoskeletal System; N: Nervous System; R: Respiratory System; S: Sensory Organs. (**B**) Main Psychotropic Drugs Prescribed in the Study Population. The figure includes only medications with more than ten prescriptions.

Figure 4 shows the distribution of prescribed drugs in the 333 patients according to the Anatomical Therapeutic Chemical (ATC) classification. Most medications belonged to the Nervous System (N) category, with 1149 prescriptions, reflecting the psychiatric profile of the study population (Figure 4A). The analysis was restricted to drugs prescribed in at least 20% of patients, with clonazepam, lormetazepam and olanzapine being the most frequently represented. In addition, drugs such as levomepromazine, trazodone, diazepam and fluoxetine were prescribed in approximately 10% of patients, among others (Figure 4B). In contrast, drugs from the Digestive Tract and Metabolism (A) category (179 prescriptions) and the Cardiovascular System (C) category (68 prescriptions) were less frequent, while the remaining categories accounted for a markedly smaller proportion of prescriptions.

**Figure 5:**
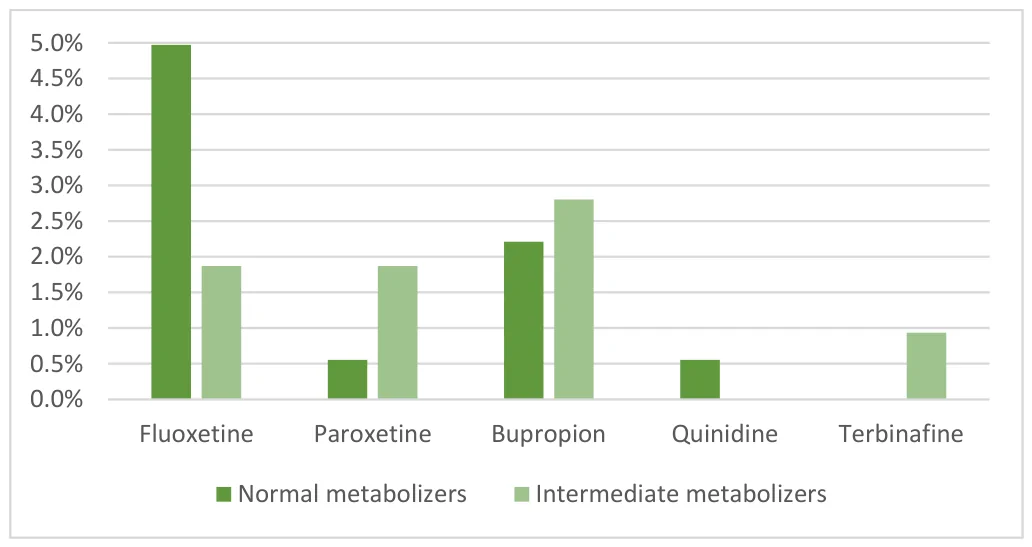
Potential drug–gene interactions (DGIs) related to CYP2D6 inhibition. Percentage of patients at possible risk of drug–gene interactions associated with the use of CYP2D6 inhibitors, based on the subset of the population identified as normal and intermediate metabolizers. No cases were identified among ultrarapid metabolizers.

After identifying the most commonly prescribed medications, all patients with available information on concomitant medication were reviewed in order to assess potential drug–gene interactions (DGIs) involving genes related to antipsychotic metabolism, specifically *CYP2D6* and *CYP3A4* [98,99,100]. Patients with normal, intermediate and ultrarapid metabolizer phenotypes for CYP2D6 were selected, as these groups are most susceptible to the endophenotype phenomenon. In these cases, metabolic activity may differ significantly from that expected based on genotype due to enzyme inhibition and induction interactions. It was observed that 8% of normal metabolizers are at possible risk of DGIs when potent CYP2D6 inhibitors are considered, with fluoxetine being the medication associated with the highest number of such interactions (Figure 5). Regarding *CYP3A4*, one case was identified in which the patient was receiving ketoconazole, a potent CYP3A4 inhibitor. Additionally, two patients were found to be undergoing treatment with CYP3A4 inducers—specifically carbamazepine and phenytoin. Notably, one of these two patients was being treated with both inducers simultaneously.

### 3.3. Pharmacogenetic Profile

**Figure 6:**
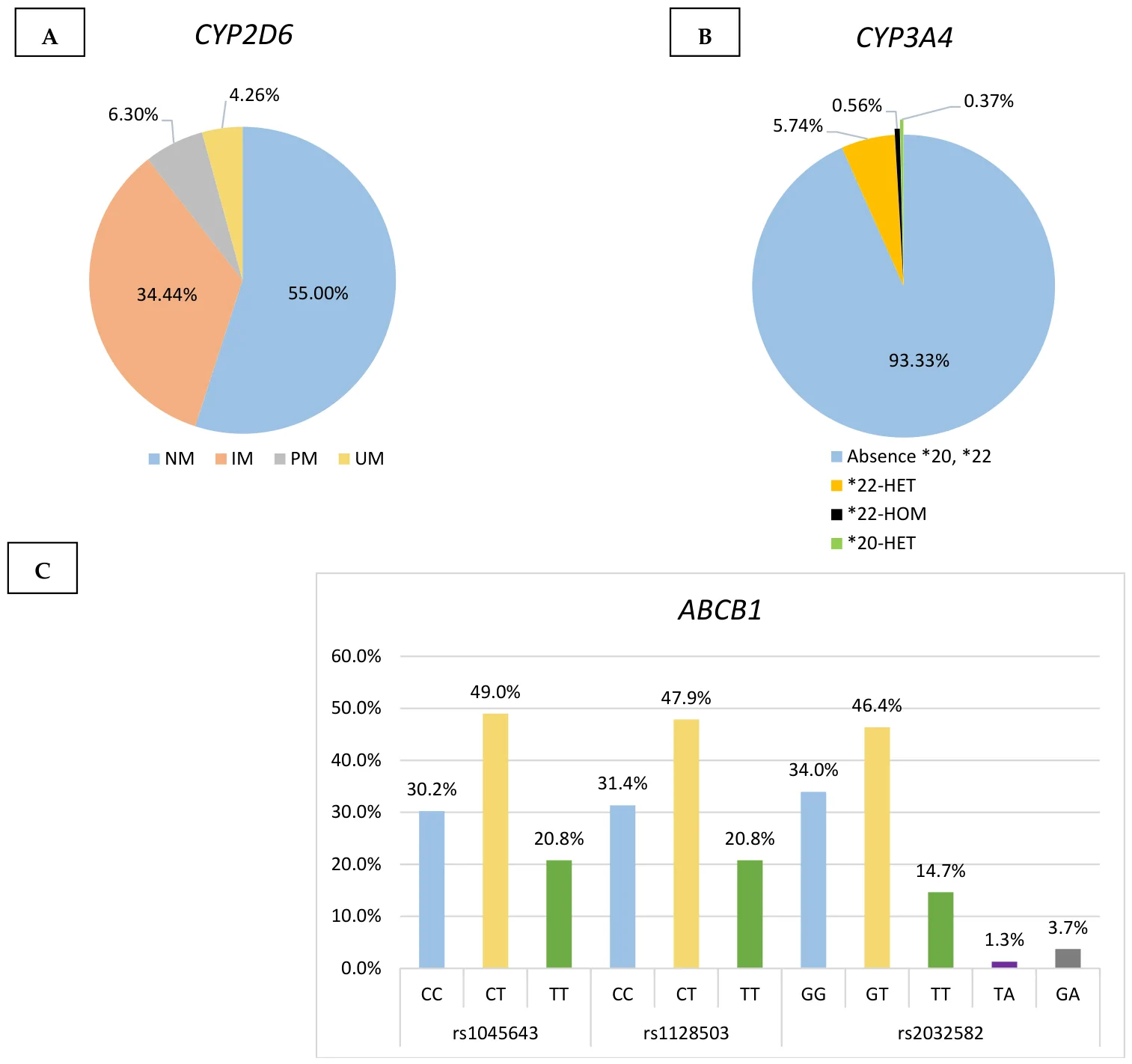
Distribution of phenotypic profiles of patients. (**A**) Distribution of *CYP2D6* metabolizer phenotypes, classified as normal metabolizers (NM), intermediate metabolizers (IM), poor metabolizers (PM) and ultrarapid metabolizers (UM). (**B**) Distribution of functional variants in *CYP3A4*, showing the absence of *20 and *22 alleles, as well as their presence in heterozygous (*20-HET, *22-HET) and homozygous (*22-HOM) states. (**C**) Genotype frequencies of ABCB1 variants rs1045642, rs1128503 and rs2032582, showing CC, CT and TT genotypes. The T allele, present in CT and TT genotypes, has been associated with reduced P-glycoprotein activity.

Pharmacogenetic analyses were performed for 540 patients. For *CYP2D6*, the distribution of predicted metabolizer phenotypes was as follows: normal metabolizers (NM) 55% (*n* = 297), intermediate metabolizers (IM) 34.44% (*n* = 186), poor metabolizers (PM) 6.30% (*n* = 34) and ultrarapid metabolizers (UM) 4.26% (*n* = 23). For *CYP3A4*, most patients showed absence of *20 and *22 alleles (93.33%, *n* = 504) with 0.56% (*n* = 3) exhibiting homozygosity for *22, while heterozygosity was observed in 0.37% (*n* = 2) for *20 and 5.74% (*n* = 31) for *22 of the cases. No cases of homozygosity for *20 were detected. Regarding *ABCB1*, the results represent the presence of the T allele for the three analyzed variants (rs1045642, rs1128503 and rs2032582) which is associated with a decreased activity of the enzyme. For rs1045642, CT was the most frequent genotype (49%), with TT at 20.8%. Similarly, for rs1128503, CT predominated (47.9%) and TT was 20.8%. For rs2032582, GT was most common (46.4%) and TT occurred in 14.7% of individuals. Additionally, the presence of the T allele was also detected in combination with the other alternative allele A, as this position is triallelic, accounting for 1.3% of the cases. The homozygous combination for the three variants (TT–TT–TT) was detected in 10.5% (*n* = 57) of patients (Figure 6).

### 3.4. Pharmacokinetic Results

Of the total cohort of 540 patients included in the Pilot Program, therapeutic drug monitoring data were available for 343 patients (Table 3). As pharmacogenetic testing and TDM were performed within routine clinical practice according to clinical needs, not all patients had both types of data available. Therefore, the following pharmacokinetic analyses were conducted on the subset of patients with available TDM results. It is important to note that, in certain patients, kinetic drug monitoring was performed at up to four time points; however, only the measurement obtained concurrently with the genetic determination was included in the present analysis These patients accounted for 350 antipsychotic determinations, as some individuals had more than one determination corresponding to different antipsychotics. For paliperidone 62.6% of determinations (*n* = 87) were within the reference range, 14.4% (*n* = 20) were below and 21.6% (*n* = 30) were above the recommended range, while 1.4% (*n* = 2) could not be assessed. For risperidone a 51.7% (*n* = 45) were within range, 19.5% (*n* = 17) were below and 27.6% (*n* = 24) were above the therapeutic range, with 1.2% (*n* = 1) not assessable. Finally, for aripiprazole a 66.9% of determinations (*n* = 83) were within the therapeutic range, 21% (*n* = 26) below, 8.9% (*n* = 11) above and 3.2% (*n* = 4) not assessable.

It should be noted that the sample size for each drug differs from the total number of treated patients described in Table 4. This variation arises because, in cases of polytherapy, pharmacokinetic data were not always available for all co-administered antipsychotics. Consequently, the number of determinations reflects the availability of plasma concentration data, which may correspond to one or both drugs per patient.

## 4. Discussion

The Pharmacogenetics Implementation Pilot Program in Psychiatry has been developed in Galicia to address an unmet clinical need in psychiatric care. The program represents a pioneering initiative by specifically addressing the implementation of pharmacogenetic and pharmacokinetic analysis of LAI antipsychotics into clinical care, and was launched as one of the first initiatives in Spain [70,80]. It is worth highlighting that the inclusion criteria of the plan have been designed to be broad and oriented to any patient who is receiving or is about to initiate treatment with any type of LAI antipsychotic, as well as those who have developed adverse reactions. The main reason was to explore different scenarios where the use of PGx and/or PK might be useful, as well as capture pharmacogenetic variability in our population.

In line with this implementation strategy, the pharmacogenetic panel was intentionally focused on biomarkers supported by regulatory recommendations and/or clinical pharmacogenetic guidelines, prioritizing markers with established evidence and potential clinical utility. Although pharmacodynamic genes such as *DRD2* have been extensively investigated in relation to treatment response and adverse effects, the available evidence remains heterogeneous and is not yet sufficient to support their routine clinical implementation. These biomarkers may be considered in future updates of the panel as clinical evidence continues to evolve. This approach ensured that the initial implementation panel was aligned with current evidence and recommendations, while allowing future expansion as new clinically relevant data become available.

There are initiatives in Spain and Europe that aim to translate pharmacogenetics into clinical care from different perspectives. On the one hand, population-based pharmacogenetic studies in healthy volunteers have been used to characterize genetic variability and its potential clinical implications. Within this framework, Zubiaur and colleagues (2021) recruited, through the GENOTRIAL project (PriME-PGx program), healthy individuals to study pharmacogenetic responses, with the aim of future treatment personalization [73]. Similarly, Albuquerque et al. (2013) conducted a descriptive, population-based study using data from healthy volunteers to characterize *CYP2D6* variability in the Portuguese population, providing a basis for improving drug efficacy and safety [101]. In contrast to population-based studies, other initiatives have focused on patients characterized by treatment-related difficulties. For example, Peña-Martín et al. (2022) studied psychiatric patients with high levels of polypharmacy who had experienced adverse reactions, treatment intolerance, partial response or therapeutic failure and who could potentially benefit from implementing pharmacogenetic-guided treatment in routine clinical practice with the 5SPM protocol [80]. In a similar approach, Squassina et al. (2025) [102] explored the utility of *CYP2D6* and *CYP2C19* analysis in patients with major depressive disorder who exhibited adverse reactions or lack of treatment response. Their findings highlight the potential clinical utility of pharmacogenetic testing in guiding antidepressant therapy [102]. Our pilot study combines a population-based trial to evaluate the feasibility of PGx and PK implementation in treated psychiatric patients with a targeted PGx application in patients with adverse drug reactions.

There are also studies, which follow a similar approach to ours, focused on multiple clinical scenarios and aiming to capture pharmacogenetic variability to identify cases in which genotype-guided therapy is most useful. Van der Drift et al. (2023) analyzed a patient population referred for PGx testing at the Erasmus Center, identifying the clinical scenarios in which such analyses were requested, including adverse drug reactions, lack of therapeutic response or before initiating therapy, among others [103]. Similarly, Ragia et al. (2024) analyzed the variability of *CYP2D6* and *CYP2C19* in Greek psychiatric patients, reporting that pharmacogenetic testing was requested for different reasons—adverse drug reactions, lack of therapeutic response, or preventive purposes—and highlighted that this genetic variability underscores the importance of using PGx to personalize treatments [104].

In terms of pharmacogenetic results, the Galician Pilot Program in Psychiatry reveals substantial phenotypic variability for *CYP2D6*. Of the patients in the cohort, 45% exhibited phenotypes different from normal according to CPIC guidelines [94]: 34.44% as IM, 6.3% as PM and 4.26% as UM. The frequencies of PM and UM are similar to those reported by Peña-Martín et al. (2022) with prevalences of PM (4.9%) and UM (4.3%) [80]. However, differences in NM frequencies are noteworthy and explainable since in the Peña-Martín study, patients already presented adverse drug reactions or therapeutic failure as baseline conditions while our study enrolled a broader patient population not restricted to these clinical conditions [80]. When comparing our *CYP2D6* frequencies with those reported by Zubiaur et al. (2021), similar results are retrieved with 58% of NM, 29% of IM 6% of PM and 5% of UM individuals [73]. These similarities might be explained by the fact that Zubiaur’s study also analyzed healthy volunteers, which is more similar to our approach since both are population-based pharmacogenetic studies. Albuquerque et al. (2013) reported frequency results comparable to ours, with 6.3% for PMs and 4.7% for UMs [101]. This similarity may reflect the partially shared genetic background between Galicia and Portugal, as suggested by population genetic studies of the Iberian Peninsula [105,106,107]. Similar allele frequency results were retrieved in two studies with south European populations, Ragia et al. (2024) in the Greek population, with PM and UM proportions of 3.6% and 5.5%, respectively, [104], and Squassina et al. (2025), showing proportions of *CYP2D6* of 3.2% PMs and 9% UMs in the Italian population [102].

Regarding the *CYP3A4* gene, more than 90% of patients in the Galician Pilot Plan did not carry any of the two variants analyzed (*20 and *22). These results are in line with the 92% carrying neither variant reported by Peña-Martín et al. (2022) [80]. When comparing allele frequencies for CYP3A4*20 in our study—described by Apellániz-Ruiz et al. (2015) to be 1.2% in the Spanish population, 0.37% is observed [85]. Concerning the *ABCB1* gene, heterozygous genotypes were the most frequent for the three variants analyzed: rs1045642 C/T (49%), rs1128503 C/T (47.9%) and rs2032582 G/T (46.4%). The T allele, reported to be associated with reduced P-glycoprotein transporter activity [86], was widely represented with 10% of individuals exhibiting the triplotype TT–TT–TT. These results are similar to those reported by Lalatović et al. (2023) in a Montenegro population—rs1045642 C/T 51.82%, rs1128503 C/T 46.36%, rs2032582 G/T 47.27%—with 11.8% carrying the TT–TT–TT diplotype [108]. On the other hand, Saiz-Rodríguez et al. (2018) also reported similar results for the variant rs1045642 in a Spanish population with C/T (51.4%), C/C (29.2%) and T/T (19.5%) [109].

In addition to pharmacogenetic analyses, pharmacokinetic monitoring provides complementary information for individualized treatment. Previous studies from our group have compared Alinity C and UHPLC-MS/MS methods for plasma determination of aripiprazole, risperidone, and paliperidone. Both methods showed high precision and reproducibility, with low systematic differences and consistent intra- and inter-assay results, supporting their suitability for routine clinical application [96,97]. Pharmacogenetic testing provides the predicted metabolic phenotype, whereas therapeutic drug monitoring reflects the patient’s actual drug exposure. Together, these complementary tools [26,110,111] provide clinicians with additional information that, alongside the patient’s clinical characteristics, treatment response, concomitant medication and other relevant factors, may support individualized therapeutic decision-making. The evaluation of how this information influences treatment modifications and clinical outcomes is beyond the scope of the present study and will be addressed in future research.

Finally, it is worth highlighting that some of the aforementioned studies encompass patients treated with concomitant medication, thus the role of drug–drug interactions (DDIs) should also be taken into consideration. DDIs might result in phenomena of enzymatic inhibition or induction altering the therapeutic response and producing a phenocopy (a clinical state in which the phenotype differs from what would be expected based on the genotype). The concomitant medications vary according to the study design and the population included. Eighty percent of patients in the Galician Plan received between 1 and 6 drugs, with a mean of 4 drugs per patient. Antipsychotics were the most frequently prescribed medications, reflecting the characteristics of the target population; however, benzodiazepines were the most common concomitant drugs, with clonazepam prescribed in 14% of patients. The mean number of drugs aligns with that observed by Peña-Martín et al., with a mean of 4.82 drugs per patient; however, quetiapine (6.5%) and olanzapine (5.7%) were the most commonly prescribed medications [80]. Van der Drift et al. analyzed a population referred for pharmacogenetic testing, in which citalopram was the most prescribed drug (8%) [103]. In 2021, the Department of Health and Social Care of the United Kingdom published a report addressing overprescribing within the NHS. The report analyzed the causes and the consequences in order to propose recommendations to reduce inappropriate prescriptions, improve patient safety and optimize medication use sustainably. The results showed that patients receive an average of 3.9 drugs each [112]. The variability in concomitant drugs highlights that psychiatric patients are often exposed to multiple medications, increasing the risk of DDIs, complicating clinical management, and underscoring the importance of pharmacogenetic analyses to optimize treatment and predict therapeutic outcomes.

Although pharmacogenetic and pharmacokinetic data were collected simultaneously within the Pilot Program, the present study was designed to describe its implementation and initial experience in routine clinical practice rather than to investigate genotype–drug concentration relationships. The integrated dataset generated through the program will enable future studies to evaluate the relationship between metabolizer phenotype, plasma drug concentrations, concentration-to-dose ratios, and clinical outcomes in patients receiving long-acting injectable antipsychotics.

It is worth highlighting that some limitations have been identified in the Implementation Plan for Pharmacogenomics in Psychiatry. One limitation is the lack of systematic follow-up data on changes in clinical practice. We are aware that adjustments have been performed in patient management according to the clinical problem; however these adjustments have not been evaluated in real time. Furthermore, this limitation is also related to the initial study design, which was not specifically intended to evaluate cost-effectiveness, although such an analysis would be of great interest in future research. In addition, the nature of the included units is variable: on the one hand, Acute Care Units were incorporated, where long-term patient follow-up is not conducted. In contrast, other units such as Continuity of Care teams or Mental Health Units were also included, which do allow prolonged patient follow-up. This plan has been designed to implement both PGx and PK independently. However, further investigation of the results is required to explore potential similarities between both approaches, especially taking into account concomitant medication co-administered with antipsychotics. In the long term, the goal would be to move toward models that integrate multiple variables to optimize dose adjustments, combining pharmacogenetic and pharmacokinetic information with clinical characteristics and also incorporating insights from other omics approaches prior to validation.

## 5. Conclusions

In conclusion, this pilot program represents a novel and comprehensive approach to precision psychiatry for long-acting injectable antipsychotics. This pilot program demonstrates the feasibility of integrating pharmacogenetic testing and therapeutic drug monitoring into routine psychiatric practice. The evaluation of how these tools influence treatment decisions and clinical outcomes represents the next phase of the program and will be addressed in future studies. Its successful integration into routine clinical practice was enabled by a robust implementation framework based on structured workflows, trained personnel, and integration with Galician’s unified electronic health record, ensuring that these advanced tools could be applied consistently and reliably. Finally, it is worth highlighting that this type of program underscores the value of structured pilot initiatives in demonstrating the potential for integrating novel biomarkers, including pharmacogenetic-guided tools, into the standard service portfolio, thereby supporting the expansion of precision psychiatry in routine clinical practice.

## Figures and Tables

**Table 1:** Alleles of pharmacogenetics panel.

Gene	Allele	Functionality
*CYP2D6*	*2, *35	Normal function
	*3, *4, *5, *6, *7, *31, *69	No function
	*9, *10, *17, *29, *36, *41, *59	Decreased function
	CNV ^1^	Structural variation
*CYP3A4*	*20, *22	Decreased function
*ABCB1*	435C>T, 2677T>G/A, 1236C>T	Decreased function

^1^ CNV: Copy number variation.

**Table 2:** Requirements for monitoring LAI antipsychotics. All plasma samples were therefore obtained under steady-state trough conditions to ensure comparability with the established therapeutic reference ranges.

**Paliperidone Monitoring**	
**Dispensing**	**Monitoring**
Oral	At least 5–7 days of treatment or after a dosage change
Monthly LAI (Xeplion^®^)	At least 4 doses administered (extraction prior to the 5th dose or subsequent doses)
Quarterly LAI (Trevicta^®^)	At least 4 doses administered (extraction prior to the 5th dose or subsequent doses)
Semiannual (Byannli^®^)	At least 2 doses administered (extraction prior to the 3rd dose or subsequent doses)
**Risperidone Monitoring**	
**Dispensing**	**Monitoring**
Oral	At least 5–7 days of treatment or after a dosage change
Fortnightly LAI (Risperdal Consta^®^)	At least 4 doses administered (extraction prior to the 5th dose or subsequent doses)
Monthly LAI ISM (Okedi^®^)	At least 2 doses administered (extraction prior to the 3rd dose or subsequent doses)
**Aripiprazole Monitoring**	
**Dispensing**	**Monitoring**
Oral	At least 20–25 days of treatment or after a dosage change
Quaterly LAI (Abilify maintena^®^)	At least 4 doses administered (extraction prior to the 5th dose or subsequent doses)
Monthly LAI (Abilify maintena^®^)	At least 4 doses administered (extraction prior to the 5th dose or subsequent doses)

**Table 3:** Demographic and treatment data.

Variable	Value
*Patients*	540
Average age	47.1 (±14.4)
Male	310 (57.4%)
Female	230 (42.6%)
*Treatment information*	
Paliperidone	220 (40.74%)
Risperidone	97 (17.96%)
Aripiprazole	194 (35.93%)
Combined antipsychotics	29 (5.37%)
Paliperidone + other ^1^	17 (58.62%)
Risperidone + other	22 (75.86%)
Aripiprazole + other	19 (65.52%)
*Request cause*	
Transition oral to LAI ^2^	208 (38.52%)
ADR ^3^	37 (6.85%)
Clinical follow-up	295 (54.63%)

^1^ other: one of the other antipsychotics of Pilot Plan (risperidone, paliperidone or aripiprazole). ^2^ LAI: long-acting injectable. ^3^ ADR: Adverse Drug Reaction.

**Table 4:** Classification of antipsychotics plasma levels according to guideline-based expected values.

Drug	*n*	Within Range	Below Range	Above Range	Not Assessable
PAL	139	87 (62.6%)	20 (14.4%)	30 (21.6%)	2 (1.4%)
RISP	87	45 (51.7%)	17 (19.5%)	24 (27.6%)	1 (1.2%)
ARI	124	83 (66.9%)	26 (21%)	11 (8.9%)	4 (3.2%)

PAL: Paliperidone; RISP: Risperidone; ARI: Aripiprazole.

## Data Availability

The data analyzed in this study are subject to the following licenses/restrictions: Data were derived from clinical practice. Requests to access these datasets should be directed to Olalla Maroñas, olalla.maronas@usc.es.

## Abbreviations

The following abbreviations are used in this manuscript:

1+ MG	1+ Million Genomes Initiative
5SPM	Five-step precision medicine model
9-OH-R	9-OH-risperidone
ACIS	Galician Agency for Health Knowledge
AEMPS	Spanish Agency for Medicines and Medical Devices
ATC	Anatomical Therapeutics Chemical
CPIC	Clinical Pharmacogenetics Implementation Consortium
DDIs	Drug–drug interactions
DHA	Dehydroaripiprazole
DPWG	Dutch Pharmacogenetics Working Group
EMA	European Medicines Agency
ESPT	European Society of Pharmacogenomics and Personalised Therapy
FDA	Food and Drug Administration
FPGMX	Public Galician Foundation of Genomic Medicine
IANUS	Electronic healthcare record
IMPaCT	the Infrastructure for Precision Medicine associated with Science and Technology
LAI	Long-acting injectable
P-gp	P-glycoprotein
PGRN	Pharmacogenomics Research Network
PGx	Pharmacogenetics
PK	Pharmacokinetic
PPM	Personalized Precision Medicine
SEFF	Spanish Society of Pharmacogenetics and Pharmacogenomics
TDM	Therapeutic drug monitoring
UHPLC-MS/MS	Ultra-high-performance liquid chromatography–tandem mass spectrometry
U-PGx	Ubiquitous Pharmacogenomics Consortium
